# How often is a low Apgar score the result of substandard care during labour?

**DOI:** 10.1111/j.1471-0528.2010.02565.x

**Published:** 2010-04-21

**Authors:** S Berglund, H Pettersson, S Cnattingius, C Grunewald

**Affiliations:** a Department of Clinical Science and Education, Karolinska Institutet SödersjukhusetStockholm, Sweden; b Clinical Epidemiology Unit, Department of Medicine, Karolinska InstitutetStockholm, Sweden

**Keywords:** Asphyxia, delivery, fetal surveillance, hyperstimulation, labour, oxytocin, substandard care, vacuum extraction.

## Abstract

**Objective:**

To increase our knowledge of the occurrence of substandard care during labour.

**Design:**

A population-based case–control study.

**Setting:**

Stockholm County.

**Population:**

Infants born in the period 2004–2006 in Stockholm County.

**Methods:**

Cases and controls were identified from the Swedish Medical Birth Register, had a gestational age of ≥33 complete weeks, had planned for a vaginal delivery, and had a normal cardiotocographic (CTG) recording on admission. We compared 313 infants with an Apgar score of <7 at 5 minutes of age with 313 randomly selected controls with a full Apgar score, matched for year of birth.

**Main outcome measure:**

Substandard care during labour.

**Results:**

We found that 62% of cases and 36% of controls were subject to some form of substandard care during labour. In half of the cases and in 12% of the controls, CTG was abnormal for ≥45 minutes before birth. Fetal blood sampling was not performed in 79% of both cases and controls, when indicated. Oxytocin was provided without signs of uterine inertia in 20% of both cases and controls. Uterine contractions were hyperstimulated by oxytocin in 29% of cases and in 9% of controls, and the dose of oxytocin was increased despite abnormal CTG in 19% and 6% of cases and controls, respectively. Assuming that substandard care is a risk factor for low Apgar score, we estimate that up to 42% of the cases could be prevented by avoiding substandard care.

**Conclusions:**

There was substandard care during labour of two-thirds of infants with a low Apgar score. The main reasons for substandard care were related to misinterpretation of CTG, not acting on an abnormal CTG in a timely fashion and incautious use of oxytocin.

## Introduction

A small, but nonetheless too large, proportion of patients experience adverse events in the hands of healthcare workers. The frequency of substandard care during labour in Sweden is unknown, as is the number of infants injured at birth as a result of substandard care, with the exception of the 20–50 annual claims for financial compensation relating to severely asphyxiated infants.^1^,^2^

In infants considered to have suffered from severe asphyxia as a result of substandard care during labour, we have previously found the most common causes to be: neglecting to supervise fetal wellbeing, neglecting signs of fetal asphyxia, incautious use of oxytocin and choosing a non-optimal mode of delivery.^1^ Several recognised risk factors for asphyxia have also been found to be associated with asphyxia related to substandard care during labour.^2^–^4^ Based on these results, we hypothesised that infants with a low Apgar score are more frequently subjected to substandard care during labour than infants with a full Apgar score.

To increase our knowledge of the occurrence of substandard care during labour, we have investigated factors related to substandard care and the risk of a low Apgar score at 5 minutes. With the overall aim of improving the safety of patients during labour, we have more specifically focused on factors related to the following points.

1. Neglecting to supervise fetal wellbeing.
2. Improper use of oxytocin.
3. Neglecting signs of fetal asphyxia.
4. Substandard care during delivery.

## Methods

We designed a population-based case-control study including the seven delivery units of Stockholm County, encompassing around 24% (*n* = 74 539) of all births in Sweden (*n* = 309 140) during the years 2004–2006. Prematurity is one of the most important risk factors for developing cerebral palsy.^5^ We chose to include only infants with a gestational age of at least 33 completed weeks, which is the time point when immaturity of the lungs is no longer treated with betamethasone. The gestational age was defined by a second-trimester ultrasound, and the infants were identified by the Swedish Medical Birth Register. As low Apgar score correlates well with asphyxia, and is, as opposed to acid–base balance, nearly always registered, cases were defined as infants born with an Apgar score of <7 at 5 minutes of age.^1^,^3^ Within our population-based cohort, we identified 415 infants with an Apgar score <7 at 5 minutes of age with a gestational age of ≥33 weeks. We excluded 102 infants because of a non-reactive cardiotocogram (CTG) on admission (*n* = 60), missing case records or CTG tracings (*n* = 31), precipitate deliveries (*n* = 7), or lethal malformations (*n* = 4). This resulted in 313 cases being used in the study. For each case, we randomly selected a healthy control with full Apgar score (10) at 5 minutes of age and matched for year of birth. Both cases (*n* = 313) and controls (*n* = 313) had planned for a vaginal delivery and had normal CTG tracing on admission to the delivery unit.

### Data collection

Data was collected similarly for cases and controls. We retrieved data from the standardised antenatal and obstetric records used throughout Sweden, CTG recordings from during labour, and the neonatal case records. Diagnoses during pregnancy and delivery were registered at discharge from the hospital, and were classified according to the tenth Swedish version of the International Classification of Diseases (ICD-10). The following information on pregnancy complications was included: pre-eclampsia (ICD-10 codes 011, 014 and 015), pregestational diabetes (ICD-10 codes 024.0–024.3) and gestational diabetes (ICD-10 code 024.4). For delivery complications, we included information on dystocia (ICD-10 code 062) and breech delivery (ICD-10 codes 064.1, 083.0 and 083.1).

Information about the date and time of birth, sex, birthweight, Apgar scores at 1, 5 and 10 minutes, umbilical cord pH, and acts of resuscitation were retrieved from the neonatal records. Birthweight for gestational age was based on the Swedish reference curve for intrauterine growth,^4^ and was defined as the ratio of observed to expected birthweight for gestational age and sex. A normal birthweight ratio was defined as a birthweight for gestational age of <10% below or above the expected weight; a moderately small birthweight ratio was defined as 10–25% below the expected weight; a very small birthweight ratio was defined as >25% below the expected weight; a moderately large birthweight ratio was defined as 10–25% above the expected weight; and a very large birthweight ratio was defined as >25% above the expected weight. The information on the degree of hypoxic ischaemic encephalopathy (HIE) was retrieved from the neonatal case records (ICD10-codes P91.0, P91.6, P91.6X, P91.6A, P91.6B and P91.6C).

According to Swedish standards, the minimum level of fetal surveillance in healthy women with uncomplicated deliveries was a CTG performed upon admission to the maternity unit for at least 20 minutes, and thereafter every other hour during the first stage of labour, assuming a normal CTG tracing.^6^ Meanwhile, the midwife should register fetal heart sounds every 15 minutes before, during and after contractions. During the first 45 minutes of the second stage of labour, the midwife should register fetal heart sounds every fifth minute; thereafter, continuous CTG monitoring is required.^6^,^7^ CTG tracing was defined as normal, intermediary or abnormal according to the International Federation of Gynecology and Obstetrics (FIGO) classifications of CTG recordings.^8^ We defined that there was an indication for fetal blood sampling (FBS) when a CTG was abnormal for more than 40–60 minutes,^6^,^7^ and that a continuous CTG recording was required during labour in the presence of maternal or fetal risk factors such as severe pre-eclampsia, pregestational and gestational diabetes, gestational age ≤36 or ≥42 completed weeks, meconium-stained amniotic fluid, suspicion of fetal growth restriction or placental insufficiency.^8^,^9^ In addition, if complications during labour occurred, such as maternal fever, bleeding, or uterine inertia treated by oxytocin, continuous CTG registration was considered compulsory. Oxytocin was administered in cases of induction of labour or after diagnosed uterine inertia, defined as no progress of labour during 2 hours after initially normal progress.^10^ After initiating the infusion of oxytocin, continuous CTG tracing with the simultaneous registration of contractions should have been performed, in conjunction with four or five contractions every 10 minutes.^6^ Hyperstimulation of uterine contractions was defined as six or more contractions every 10 minutes over a period of at least 20 minutes.^11^,^12^ If oxytocin infusion became complicated by tachysystole, oxytocin should have been discontinued or the rate of infusion decreased, at least temporarily, to avoid uterine hyperstimulation.^2^,^4^,^12^

Care during labour was considered substandard when supervision of fetal wellbeing was neglected. This included when there were no CTG recordings for more than 2.5 hours after the admission test or in between CTG recordings, and when CTG recordings were not interpretable because they were of poor quality. In cases of continuous abnormal CTG but normal FBS, a follow-up of FBS should have been carried out every 20–30 minutes, dependent on the stage of delivery.^6^ Moreover, we considered that there had been substandard care during labour when signs of fetal asphyxia were neglected, and when there was untimely action on abnormal CTG (i.e. action was taken more than 45 minutes from the onset of an abnormal CTG to birth), uterine hyperstimulation, or if the oxytocin infusion was increased despite an abnormal CTG (Box1).

**Box 1:** Neglecting to supervise fetal wellbeing and signs of fetal asphyxia

There was substandard care during labour if:
•no CTG recordings were made for more than 2.5 hours after the admission test;
•more than 2.5 hours elapsed between CTG recordings;
•CTG recordings were not interpretable because of poor quality;
•no FBS was performed despite continuous intermediary or abnormal CTG for more than 45 minutes;
•no follow-up of FBS despite continuous abnormal CTG with a normal FBS;
•no follow-up of pre-acidotic FBS (according to stage of delivery);
•untimely action on abnormal CTG (i.e. more than 45 minutes from onset of abnormal CTG to birth);
•uterine tachysystole (more than six contractions every 10 minutes for more than 20 minutes);
•increased dose of oxytocin despite abnormal CTG or tachysystole.

We defined imminent asphyxia as an FBS with a pH of <7.20, a lactate value of >4.8 mmol/l, a terminal CTG, or abnormal CTG tracings with prolonged bradycardia, tachycardia, a complicated variable, or late decelerations. We considered that care was substandard during delivery in the cases of imminent asphyxia if the time from the decision to deliver to birth exceeded 30 minutes, if there was a spontaneous vaginal delivery despite longstanding (at least 45 minutes) abnormal or uninterpretable CTG recordings, or if there was a complex instrumental delivery. Complex instrumental delivery was defined as an inappropriate trial of labour in a singleton delivery with a vacuum extractor or forceps in the following circumstances: incomplete cervical dilatation, non-cephalic presentation or cephalic malpresentation, non-engaged fetal head, or a clear indication of cephalopelvic disproportion. The definition of a complex instrumental delivery also included a delivery by vacuum extraction exceeding more than 20 minutes or following more than two cup detachments (Box 2).^13^–^17^

**Box 2:** Substandard care during delivery

There was substandard care during delivery if:
•the time from the decision to deliver to birth exceeded 30 minutes in the case of imminent asphyxia;
•there was a spontaneous vaginal delivery despite a long-standing (at least 45-minute) abnormal or uninterpretable CTG recording;
•a complex vaginal instrumental delivery, defined as an inappropriate trial of labour with a vacuum extractor or forceps in the following circumstances:^13^–^17^
•incomplete cervical dilation;
•non-cephalic presentation or cephalic malpresentation;
•non-engaged fetal head;
•a clear indication of cephalopelvic disproportion;
•extraction exceeding more than 20 minutes (i.e. more than the recommended 15 minutes);
•more than two cup detachments.

## Statistics

### Sample size

We designed the study as a case-control study, frequency matched for year of delivery. A power analysis was completed before implementation. A sample size estimate of 319 patients in each group (638 in total) would provide an 80% power to detect a two-fold increase in the odds of substandard care between infants with low (cases) and full (controls) Apgar scores, with a two-sided alpha = 0.05. This calculation is based on the assumption that 10% of the controls were subjected to substandard care, and was calculated in Sample Power 2.0. Among infants born in Stockholm County in 2004–2006, there were 415 infants with a gestational age of ≥33 completed weeks that had Apgar scores of <7 at 5 minutes of age: thus, this population seemed to be sufficient for our purpose.

### Data analyses

To investigate possible risk factors for low Apgar scores, we used unconditional logistic regression analyses (Table 1). The possible risk factors for low Apgar score included: (a) maternal characteristics that are available before the woman arrives at the hospital; (b) characteristics related to care during labour; and (c) infant characteristics. For clarity of effect and clinical interpretation, continuous factors were categorised as previously described.^18^ We then used multivariable models to study the adjusted associations, i.e. maternal characteristics and characteristics related to care that were adjusted for each other, whereas infant characteristics were adjusted for other infant characteristics (Table 1).

**Table 1:** Descriptive data and risk for an Apgar score of <7 at 5 minutes of age

	Cases *n* = 313		Controls *n* = 313		Odds ratios (95% CI)	
	*n*	%	*n*	%	Unadjusted	Adjusted*
**Maternal characteristics**						
**Maternal age (years)**						
≤24	21	6.7	40	12.8	1.0	1.0
25–29	78	24.9	72	23.0	2.1 (1.1–3.8)	2.8 (1.4–5.8)
30–34	131	41.9	125	39.9	2.0 (1.1–3.6)	3.2 (1.6–6.6)
≥35	83	26.5	76	24.3	2.1 (1.1–3.8)	4.0 (1.8–8.6)
**Parity**						
0	214	68.4	148	47.3	2.9 (2.0–4.1)	2.6 (1.6–4.0)
≥1, no caesarean section	76	24.3	151	48.2	1.0	1.0
≥1, at least one caesarean section	23	7.3	14	4.5	3.3 (1.6–6.7)	2.3 (1.0–5.2)
**Infertility (years)**						
0	287	91.7	289	92.3	1.0	1.0
1	4	1.3	13	4.2	0.3 (0.1–1.0)	0.2 (0–0.6)
≥2	20	6.4	8	2.6	2.5 (1.1–5.8)	1.6 (0.6–4.1)
Data missing	2	0.6	3	1.0		
**Characteristics related to care**						
**Onset of delivery**						
Spontaneous	228	72.8	263	84.0	1.0	1.0
Induced	85	27.2	50	16.0	2.0 (1.3–2.9)	1.6 (1.0–2.5)
**Epidural**						
No	113	36.1	190	60.7	1.0	1.0
Yes	200	63.9	123	39.3	2.7 (2.0–3.8)	1.9 (1.3–2.8)
**Month of birth**						
1 Jan–15 Jun	174	55.6	156	49.8	1.4 (1.0–2.1)	1.5 (1.0–2.3)
16 Jun–15 Aug	59	18.8	51	16.3	1.5 (1.0–2.5)	1.4 (0.8–2.4)
16 Aug–31 Dec	80	25.6	106	33.9	1.0	1.0
**Mode of delivery and risk of an Apgar of <7**						
Spontaneous vaginal	124	39.6	269	85.9	1.0	1.0
Vacuum extraction	108	34.5	25	8.0	9.4 (5.8–15.2)	7.5 (4.3–12.8)
Forceps	5	1.6	1	0.3	10.8 (1.2–93.8)	6.8 (0.7–63.4)
Caesarean section	76	24.3	18	5.8	9.6 (5.3–15.9)	7.2 (3.8–13.9)
**Infant characteristics**						
**Gestational age (weeks)**						
33 + 0–36 + 6	28	9.0	11	3.5	3.0 (1.4–6.1)	2.3 (1.1–4.9)
37 + 0–41 + 6	240	76.7	282	90.1	1.0	1.0
≥42	45	14.4	20	6.4	2.6 (1.5–4.6)	2.7 (1.5–4.7)
Birthweight for gestational age**						
Very small	12	3.8	3	1.0	5.0 (1.4–18.0)	4.9 (1.3–18.3)
Moderately small	65	20.8	59	18.8	1.4 (0.9–2.1)	1.3 (0.8–2.0)
Normal	160	51.6	200	63.9	1.0	1.0
Moderately large	65	20.8	43	13.7	1.9 (1.2–2.9)	1.9 (1.2–3.0)
Very large	8	2.6	8	2.6	1.2 (0.5–3.4)	1.2 (0.4–3.4)
Missing	3	1.0				
**Sex of infant**						
Male	190	60.7	153	48.9	1.6 (1.2–2.2)	1.5 (1.1–2.1)
Female	123	39.3	160	51.1	1.0	1.0
**Presentation**						
Occiput anterior	282	90.1	301	96.2	1.0	1.0
Other cephalic***	24	7.7	10	3.2	2.6 (1.2–5.4)	2.7 (1.2–5.9)
Breech	7	2.2	2	0.6	3.7 (0.8–18.1)	2.4 (0.4–13.4)
**Simplex or duplex**						
Simplex	300	95.8	310	99.0	1.0	1.0
Duplex	13	4.2	3	1.0	4.5 (1.3–15.9)	2.6 (0.6–10.4)

* Analysis of maternal characteristics and characteristics related to care are adjusted for both maternal characteristics and characteristics related to care, and is based on 264 cases and 268 controls. Analyses of infant characteristics are adjusted for infant characteristics, and are based on 310 cases and 313 controls.

** Definitions are included in the Methods section.

*** Occiput posterior, forehead or facial presentation.

Next, we investigated the associations between factors reflecting substandard care and risk of an Apgar score of <7 at 5 minutes (Tables 2 and 3). In the multivariable analyses, we adjusted for maternal care and infant characteristics that were significantly (*P* < 0.05) associated with an Apgar score of <7 at 5 minutes in the univariable models in Table 1 (except mode of delivery), and added each factor related to substandard care one at a time (Tables 2 and 3). The associations are presented as odds ratios (ORs) with 95% confidence intervals (CIs). The Hosmer–Lemeshow goodness-of-fit test was used to examine if the models adequately fitted the data, and a *P* value > 0.05 indicates an acceptable fit.

**Table 2:** Substandard care during labour and risk of an Apgar score of <7 at 5 minutes of age

	Cases *n* = 313		Controls *n* = 313		Odds ratios (95% CI)	
	*n*	%	*n*	%	Unadjusted	Adjusted*
Any substandard care** during labour and risk of an Apgar of <7						
No event of substandard care	118	37.7	202	64.5	1.0	1.0
Any event of substandard care	195	62.3	111	35.5	3.0 (2.1–4.1)	2.6 (1.8–3.8)
**Intermittent CTG despite clear indication for continuous use**						
Correct intervals of CTG	288	92.0	289	92.3	1.0	1.0
Intermittent despite indication for continuous use of CTG	25	8.0	24	7.7	1.0 (0.6–1.9)	1.0 (0.5–2.0)
Abnormal CTG before birth and risk of an Apgar of <7***						
Normal CTG	67	21.4	207	66.1	1.0	1.0
Abnormal CTG, <45 minutes before birth	94	30.0	67	21.4	4.3 (2.9–6.6)	4.2 (2.6–6.8)
Abnormal CTG, 45–90 minutes before birth	65	20.8	23	7.3	8.7 (5.0–15.1)	7.6 (4.0–13.9)
Abnormal CTG, ≥90 minutes before birth	87	27.8	16	5.1	16.8 (9.2–30.6)	15.1 (7.6–30.1)
**Indication for fetal blood sampling (FBS) and risk of an Apgar of <7**						
No indication for FBS	157	50.2	279	89.1	1.0	1.0
FBS when indicated**** performed	33	10.5	7	2.2	8.4 (3.6–19.4)	7.3 (3.0–17.9)
FBS when indicated**** not performed	123	39.3	27	8.6	8.1 (5.1–12.8)	7.2 (4.3–12.0)
**Use of oxytocin, signs of uterine inertia, hyperstimulation of contractions and risk of an Apgar of <7**						
*No oxytocin*	67	21.4	151	48.3	1.0	1.0
*Oxytocin*						
No hyperstimulation,***** no inertia	22	7.1	31	10.1	1.6 (0.9–3.0)	0.9 (0.4–1.9)
No hyperstimulation, inertia	83	26.9	59	19.2	3.2 (2.0–4.9)	1.8 (1.0–3.2)
Hyperstimulation,***** no inertia	26	8.4	12	3.9	4.9 (2.3–10.2)	3.4 (1.4–7.8)
Hyperstimulation,****** inertia	63	20.4	15	4.9	9.5 (5.0–17.8)	5.5 (2.6–12.0)
No registration of contractions, no inertia	24	7.8	25	8.0	2.2 (1.2–4.1)	1.8 (0.9–3.6)
No registration of contractions, inertia	28	9.1	20	6.5	3.2 (1.7–6.0)	2.1 (1.0–4.4)
**Increase of intravenous oxytocin despite abnormal CTG and risk of an Apgar of <7**						
No	255	81.5	295	94.2	1.0	1.0
Yes	58	18.5	18	5.8	3.7 (2.1–6.4)	3.3 (1.8–6.0)

* Adjusted for the following significant variables in the univariable analyses in Table 1: maternal age, parity, infertility, onset of delivery, epidural, season of birth, gestational age, birthweight, sex of infant, and simplex/duplex. Analysis based on 308 cases and 310 controls. Hosmer and Lemeshow: *P*= 0.05–0.99.

** Any definition of substandard care, as described in Boxes 1 and 2.

*** Fetal surveillance with STAN was used in five cases and four controls. All five cases had abnormal CTG tracings between 45 and 90 minutes, of which three had hyperstimulation of contractions and no STAN events. One of these deliveries was further complicated by the rupture of the uterus. All four controls had normal CTG throughout labour without STAN events.

**** Including follow-up of FBS when indicated: yes, follow up, cases *n* = 33, controls *n* = 7; no, follow-up, cases *n* = 32, controls *n* = 12.

***** Defined as six contractions every 10 minutes for a period of more than 20 minutes.

****** Of which three ocurred after induction with prostaglandins.

We chose to divide the year into three seasons – spring, summer and autumn – to investigate if there were differences in the risks of being born in the spring season, which is the busiest time period of the year, or in the summer, when there could be a shortage of skilled obstetricians and midwives because of holidays, compared with being born in the autumn (Table 1).

As all CTG tracings were scrutinised by only one of the authors (S.B.), we independently investigated the interobserver agreement between S.B. and an expert within the field (Professor Ingemar Ingemarsson, I.I., a senior consultant). One hundred randomly selected intrapartal CTG tracings from the cases and controls were assessed by S.B. and I.I., and were classified as normal, intermediary or abnormal, and if the pattern changed, the time point was noted accordingly. The interobserver agreement in the assessment of CTG tracings between two senior consultants (S.B. and I.I.) was 86%, which was considered to be satisfactory.^19^,^20^

Finally, we estimated the (unadjusted) attributable risk related to any substandard care (according to Boxes 1 and 2 and Tables 2 and 3), and a corresponding 95% confidence interval, by applying the delta method. The attributable risk assumes a cause–effect relationship between exposure (substandard care) and disease (low Apgar score at 5 minutes), and provides an estimate of the proportion of cases that are possibly related to substandard care.^21^,^22^

Analyses were performed in spss 17.0 (SPSS Inc., Chicago, IL, USA). The study was approved by the Research Ethics Committee at the Karolinska Institutet, Stockholm (no. 2008/1375).

## Results

**Figure 1:**
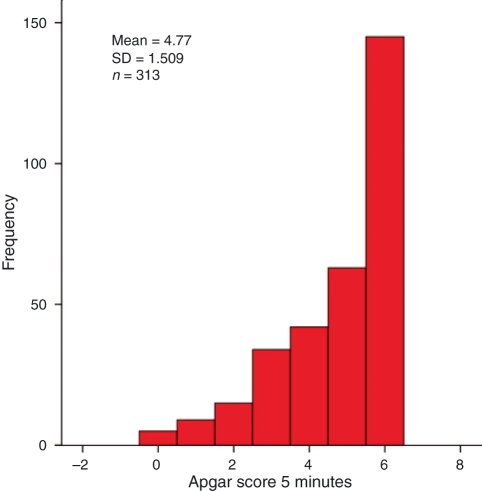
Apgar score at 5 minutes of age.

Of 313 infants with Apgar scores of <7 at 5 minutes of age (Figure 1), 90 (47%) also had an Apgar score of <7 at 10 minutes. Sixty-two cases (20%) were assessed to have HIE, of which 25 infants had HIE I and eight infants had HIE III. Eight of the 313 infants died during the neonatal period.

The risk of a low (<7) Apgar score at 5 minutes was increased among infants to mothers aged 25 years or older, compared with younger mothers (Table 1). Primiparous women, as well as women with a previous caesarean section, had a more than doubled risk compared with parous women without a previous caesarean section. Women with infertility of ≥2 years had an increased risk in the crude but not in the adjusted analysis, whereas woman with 1 year of stated infertility appeared to have a reduced risk. There were no differences between cases and controls in smoking habits, family situation, mother’s country of birth, maternal height, body mass index (BMI), hypertension, or pregestational or gestational diabetes (data not shown).

Characteristics related to care significantly associated with increased risk of a low Apgar score were induction of labour and use of epidural (Table 1). The risk of a low Apgar score was not influenced by season of birth, hour of birth, location of maternity hospital, or day of delivery (weekdays versus weekends).

Compared with term (37–41 weeks of gestation) infants, both preterm (33–36 weeks of gestation) and post-term (≥42 weeks of gestation) infants had a more than doubled risk of low Apgar score. Very small for gestational age infants faced a five-fold increase in risk, and moderately large for gestational age infants had a doubled risk of a low Apgar score, compared with normal weight infants. Boys had a 50% increase in risk compared with girls, and infants in other than occiput anterior presentation had a more than doubled increase in risk compared with infants presented in occiput anterior position (Table 1).

Forty percent (*n* = 124) of infants with an Apgar score of <7 at 5 minutes were delivered spontaneously vaginally, versus 86% (*n* = 269) of the healthy controls. Vacuum extraction was performed in 35% (*n* = 108) of cases versus 8% (*n* = 25) of the controls, delivery by forceps was performed in 1.6% (*n*= 5) of cases versus 0.3% (*n* = 1) of controls, and delivery by caesarean section was performed in 24% (*n* = 76) of cases versus 6% (*n* = 18) of controls. Infants born instrumentally or abdominally had a seven-fold increase in the risk of an Apgar score of <7 at 5 minutes compared with infants born spontaneously vaginally (Table 1).

Table 2 shows associations between signs of substandard care during labour and risks of a low Apgar score at 5 minutes. Almost two-thirds of the cases and one-third of the controls were assessed as being subjected to some form of substandard care during labour. If substandard care was present, there was an almost three-fold increased risk of low Apgar score at 5 minutes. Fetal heart rate (FHR) was assessed with CTG in most cases and controls. All CTG tracings were of good quality and were interpretable, and there were additional CTG tracings performed after the admission test in most cases (*n* = 306) and controls (*n* = 297). The interval between the last CTG tracing and birth only exceeded 2.5 hours in four cases and seven controls. There were no differences between groups concerning continuous and intermittent supervision of CTG.

Twenty-one percent of the infants with an Apgar score of <7 at 5 minutes and 66% of the controls had normal CTG tracings throughout labour. Compared with labours with normal CTG tracings, labours with abnormal CTG duration of less than 45 minutes, of 45–90 minutes, and of 90 minutes or more, had a four-fold, seven-fold, and almost 15-fold increased risk for an Apgar score of <7 at 5 minutes, respectively. FBS was indicated in 156 cases and 34 controls, but was only performed in 33 cases and seven controls. Compared with labours without an indication for FBS, there was a seven-fold increased risk of low Apgar score at 5 minutes for labours with an indication of FBS, irrespective of whether it was performed or not.

Oxytocin during labour was used in almost 80% of the cases and in 52% of the controls. Despite no signs of inertia, oxytocin was provided in more than 20% of the cases and controls, respectively. Compared with labours without uterine inertia and without the use of oxytocin during labour, the risk of a low Apgar score was doubled if there was uterine inertia and oxytocin was provided despite no signs of hyperstimulation. If the use of oxytocin resulted in hyperstimulation, the magnitude of the risk of a low Apgar score was dependent on whether there was uterine inertia (OR 5.6) or not (OR 3.4). Providing oxytocin during labour without simultaneous registration of contractions more than doubled the risk of asphyxia, and if the dose of oxytocin was increased despite abnormal CTG, the risk of asphyxia was increased by more than three-fold (Table 2).

Table 3 illustrates substandard care during delivery and the risk of a low Apgar score. Threatening asphyxia during labour was assessed in 62% (*n* = 193) of cases and in 15% (*n* = 47) of controls. When threatening asphyxia was assessed, there was a more than seven-fold increased risk of an Apgar score of <7 at 5 minutes. In pregnancies with threatening asphyxia and a decision for delivery, the risk was greater for those being delivered within 30 minutes from decision (OR 8.5) than for those being delivered after 30 minutes or more (OR 5.4). Compared with spontaneous vaginal deliveries with normal CTG throughout labour, the risk of a low Apgar score was more than doubled in pregnancies where the CTG recording had been abnormal for less than 45 minutes, and increased more than seven-fold if the CTG recording had been abnormal for more than 45 minutes. Infants with a complex instrumental delivery faced an eighteen-fold increased risk of asphyxia compared with infants with non-complex modes of delivery (Table 3).

**Table 3:** Substandard care during delivery and risk for an Apgar score of <7 at 5 minutes of age

	Cases *n* = 313		Controls *n* = 313		Odds ratios (95% CI)	
	*n*	%	*n*	%	Unadjusted	Adjusted*
**Imminent asphyxia and decision to deliver interval**						
No Imminent asphyxia	120	38.3	266	85.0	1.0	1.0
Birth within 30 minutes**	132	42.2	28	8.9	10.5 (6.6–16.6)	8.5 (5.0–14.3)
Birth after 30 minutes**	42	13.4	11	3.5	8.5 (4.2–17.0)	5.4 (2.4–11.8)
Point of time not noted	19	6.1	8	2.6	5.3 (2.2–12.4)	5.0 (1.9–13.2)
**Spontaneous vaginal delivery and risk of an Apgar of <7 in relation to abnormal CTG**						
Normal CTG	44	14.1	185	59.1	1.0	1.0
Abnormal for ≤45 minutes	39	12.5	61	19.5	2.7 (1.6–4.5)	2.6 (1.4–4.8)
Abnormalfor >45 minutes	41	13.1	23	7.3	7.5 (4.1–13.8)	7.2 (3.6–14.7)
Not spontaneous vaginal	189	60.4	44	14.1		
Complex instrumental delivery***						
No	284	90.7	313	100	1.0	1.0
Yes	29	9.3	0	0	25.4 (6.1–106.)	17.7 (4.1–77.1)

* Adjusted for the following significant variables in the univariable analyses in Table 1: maternal age, parity, infertility, onset of delivery, epidural, season of birth, gestational age, birthweight, sex of infant, simplex/duplex and presentation. Analysis based on 308 cases and 310 controls. Hosmer and Lemeshow: *P*= 0.11–0.64.

** Minutes from the noted time point of decision to birth.

*** Including inadequate trial of labour *n* = 16; >20 minutes of traction *n* = 10; more than two cup detachments *n* = 3.

The estimated unadjusted attributable risk for any substandard care was 0.42 (95% CI 0.30–0.54). Thus, assuming that substandard care (according to Boxes 1 and 2 and Tables 2 and 3) is causative for a low Apgar score, and that the prevalence of substandard care in our randomly selected control sample is a good approximation of the prevalence of the population, we estimate that up to 42% of the cases with a low Apgar score were possibly attributable to substandard care.

## Discussion

In this population-based case-control study from Stockholm County, Sweden, we found substandard care not only in 62% of infants who had an Apgar score of <7 at 5 minutes, but also in 36% of controls. Similarly to other reports, the main reasons for substandard care were related to the misinterpretation of CTGs, not acting in a timely fashion upon an abnormal CTG and incautious use of oxytocin,^1^,^2^,^23^ which are factors that, in theory, are possible to prevent, and in practice should be possible to reduce. Assuming a causal association between substandard care and low Apgar scores, we estimate that up to 42% of all cases could be prevented if substandard care during labour was avoided.

Even if infants recover neonatally without visible sequelae after signs of asphyxia at birth, we still do not know much about long-term prognosis. In a recently published study, it was shown that infants, who after resuscitation remained healthy during the neonatal period, had an increased risk of low IQ scores later in life.^24^ In line with these findings, in a follow-up study of the adolescence of 43 infants with moderate HIE, Lindstrom *et al.*^25^ found cognitive dysfunction that interfered with their daily lives in almost 80% of cases, irrespective if they had developed cerebral palsy or not.

After more than 30 years of the routine use of CTG, the most common reason for substandard care during labour is related to insufficiencies in fetal surveillance.^1^,^23^,^26^ Almost 50% of the cases and 12% of controls had abnormal CTG tracing for more than 45 minutes before birth. The longer the CTG was abnormal, the greater was the risk of a low Apgar score. If CTG was abnormal for more than 90 minutes before birth, the risk was increased by fifteen-fold compared with deliveries with normal CTG tracings. Moreover, despite the indication for continuous CTG recording, intermittent CTG registration was performed in 8% of cases and controls, which also speaks in favour of further educational efforts relating to risk factors for asphyxia and fetal surveillance.^8^,^11^,^18^,^27^,^28^

The introduction of lactate analysis in scalp blood has simplified the method of FBS, as only 5 μl of blood is required for lactate determination, in contrast to the larger volume needed for pH analysis.^29^ Nevertheless, FBS has fallen into disrepute for being a complicated, uncomfortable and time-consuming procedure, and because far more time than expected is spent from the decision to perform FBS to delivery in cases of acidaemia.^30^,^31^ When there was an indication for FBS, it was not performed in 80% of both cases and controls, and there was a seven-fold increase in risk of a low Apgar score, even if FBS was performed. These results emphasise the importance of reacting promptly to abnormal CTGs, and to the importance of a skilled and cooperative team working together in these situations.

We acknowledge that the prerequisites and guidelines for fetal surveillance differ around the world. The use of CTG is controversial, and its efficacy is being questioned.^32^,^33^ However, according to Swedish standards, CTG and FBS are the main tools for the assessment of fetal wellbeing during labour, and it is expected that they will be used.^6^,^34^

The finding of no difference in risk for asphyxia around the clock in the present study differs from a previous report encompassing deliveries from the whole of Sweden, where we found a doubled risk for asphyxia in children born at night.^18^ Nor was there a clearly increased risk associated with being born in the spring season, which is characterised by the largest number of births throughout the year, and periodically results in a shortage of delivery rooms in Stockholm County. Our findings may be explained by the fact that all maternity units in Stockholm are similarly operated around the clock, and that skilled staff can be in place within 30 minutes.

Incautious use of oxytocin can contribute to asphyxia.^1^–^4^ We found that the number of deliveries provided with oxytocin infusion was unexpectedly high in both cases and controls, and more than every fifth case and control received oxytocin despite no signs of inertia. Another misuse of oxytocin included uterine hyperstimulation, increasing oxytocin infusion despite abnormal CTG and no registration of contractions.^8^,^12^

In cases of imminent asphyxia, the international standard is to deliver within 30 minutes.^35^,^36^ We found that a short time period from decision to delivery was associated with poor neonatal outcome. Obviously, the obstetric history during labour, such as long-standing abnormal CTG, not acting in a timely fashion on signs of asphyxia, and the cause of emergency, has a great influence on the association between time from decision to delivery and the neonatal outcome.^37^

One of the hallmarks of professional midwifery is time-intensive supportive care, which has been shown to be associated with lower rates of caesarean section and fetal distress.^38^ In Sweden, midwives are independently responsible for normal deliveries, and are thereby expected to be skilled in interpreting CTGs. In the case of an intermediary or abnormal CTG tracing, the midwife is obliged to consult a physician. We found that spontaneous vaginal delivery could be hazardous in cases of an abnormal CTG, which emphasises the importance of the correct interpretation of CTG among midwives assisting normal deliveries. Similarly, in accordance with other studies, we found that sound knowledge and skills among physicians when delivering instrumentally is of great importance for neonatal outcome.^13^

Our study shows that necessary efforts to make delivery safer include infrastructural changes and institutional incentives. Aviation used to be a high-risk industry, but has been transformed by the widespread utilisation of information technology, in particular autopilots, and several mistakes must therefore be made before an accident can take place. In addition to aviation, oil and nuclear industries, the formal investigation of incidents, consisting of a series of routinely followed steps, is well established. Studies in these areas have led to a broader understanding of the causes of accidents, with less focus on the individual’s mistake and more on pre-existing organisational factors. Such studies have also illustrated the complexity of the chain of events that may lead to an adverse outcome.^39^–^42^

Every case of asphyxia can be used as a learning example. Perinatal audits consisting of professionals within perinatal care are useful when investigating shortcomings in conjunction with childbirth. The aim of an audit is hence to initiate adjustments, optimise the quality of care and improve interprofessional collaboration. Perinatal audits have been shown to be efficient in the Netherlands, where perinatal mortality in 1999 was ranked the highest (11.4/1000) among the 25 countries of the European Union.^43^,^44^ The use of clinical dashboards for monitoring and presenting regular healthcare performance is gaining popularity. Clinical dashboards may be useful in predicting trends in various clinical governance parameters, enabling immediate action to be taken to rectify patient safety issues. When a maternity dashboard was introduced at St Georges Hospital, London, in 2007, there was an ‘amber–red’ trend of increasing emergency caesarean section after failed instrumental deliveries. Immediate action was taken, with educational efforts focussed on hands-on ventouse training and a daily review on emergency caesarean sections, after which there was a decrease in the caesarean section rate from 26% to 20%.^45^–^47^

Draycott *et al.*,^48^ in Bristol, UK, have demonstrated that mandatory skills training in obstetric emergencies improves neonatal outcome. Besides, educational efforts among physicians and midwives may improve CTG interpretation and fetal surveillance.^49^ Recently, the Swedish Society of Obstetrics and Gynaecology has, in collaboration with the Swedish Association of Midwives, developed an internet-based interactive and freely available CTG educational and training programme, providing a certificate after having passed the final examination.^50^ Additionally, standardised stickers for the interpretation of CTG have been developed, and are now available on a national level. However, although there are examples of successful outcomes, such as the introduction of training and stickers, it is uncertain to what extent improvements can be achieved. In an online educational setting in CTG and acid–base interpretation in the UK, the mean score in written tests after education were around 63 and 83% for midwives and obstetricians, respectively, which indicates that additional educational efforts are needed.^49^ Questions on whether there are a certain number of people who suffer from cognitive dysfunction, resulting in pattern dyslexia, have been raised.^49^ If so, besides training and education, maybe CTG interpretation should be assessed by at least two people, or by additional computerised interpretation functions.^23^,^51^

## Conclusion

Some form of substandard care during labour was present in two-thirds of deliveries of infants with a low Apgar score at 5 minutes, and in one-third of the controls. The main reasons for substandard care were related to the misinterpretation of CTGs, not acting in a timely fashion on abnormal CTGs, and the incautious use of oxytocin. Assuming that substandard care is a risk factor for a low Apgar score, we estimate that the number of infants with an Apgar score of <7 at 5 minutes of age could be substantially reduced by preventing substandard care.

### Disclosure of interests

There are no conflicts of interests. The sponsors played no part in the study design, data analyses, data interpretation, or in the writing of the report. SB and HP had full access to all the data in the study, and the final responsibility for the decision to submit for publication was shared by all authors.

### Contribution to authorship

The study was planned by all of the authors. SB collected all the data, carried out the main part of the analyses and drafted the manuscript. CG, HP and SC assisted in the analyses, in the interpretation of the results and the revisions to the manuscript. The final version of the manuscript has been approved by all of the authors.

### Details of ethics approval

The study was approved by the Research Ethics Committee at the Karolinska Institutet, Stockholm (no. 2008/1375).

### Funding

We thank the Swedish County Council Insurance Company for financing the major part of the study. We also thank the Karolinska Institutet, Södersjukhuset, Her Majesty Queen Silvia’s Jubilee Fund, The Josef and Linnea Carlsson Foundation, Schering-Plough, Majblomman Foundation, The Elsa Goije Memorial Foundation, and The Folke Bernadotte Foundation for generous financial support.
